# Implementation of rapid COVID-19 testing at Massachusetts trial courts

**DOI:** 10.1186/s40352-023-00220-1

**Published:** 2023-04-10

**Authors:** Yvane Ngassa, Emma Smyth, Bridget Pickard, Morgan Maner, Lauren Brinkley Rubinstein, Alysse Wurcel

**Affiliations:** 1 Department of Medicine, Division of Geographic Medicine and Infectious Diseases, Tufts Medical Center, 15 Kneeland St, Boston, MA 02111 USA; 2 Brody School of Medicine, Eastern Carolina University, Greenville, NC USA; 3 Department of Population Health Sciences, Duke University, Durham, NC USA

**Keywords:** Trial court, COVID-19 testing, Justice-involved, Criminal-legal

## Abstract

**Background:**

COVID-19 shut down trial courts across the country, prolonging case resolution of charged, detained, and incarcerated people. We report on the implementation of rapid COVID-19 testing at Trial Courts in Massachusetts (MA), focusing on the outcomes of adoption and acceptability.

**Methods:**

Guided by the Expert Recommendations in Implementing Change (ERIC) framework, we chose six strategies to guide implementation. After assembling a group of stakeholders, including representatives of the Trial Court, Department of Public Health (DPH) and vendors providing COVID-19 testing, we implemented rapid COVID-19 testing at Trial Court locations in December 2021. We collected data on (1) adoption of COVID-19 testing, (2) number of stakeholders who attended meetings, (3) number of tests performed at Trial Court sites, and (4) acceptability of COVID-19 testing using a QR-code anonymous survey.

**Results:**

There was a high percentage of attendance at stakeholder meetings (> 70% at each meeting). 243 COVID-19 tests were conducted on eight occasions at four Trial Court sites between December 2021 and February 2022. Participants who responded to the QR-code survey reflected favorably on COVID-19 testing at MA Trial Court sites.

**Conclusion:**

COVID-19 testing at Massachusetts Trial Court sites was possible through stakeholder engagement. Several cases of COVID-19 were identified prior to entry into the Trial Court. Funding for rapid COVID-19 testing should be provided to help keep trial courts open as the pandemic continues to evolve.

## Introduction

Carceral settings, including but not limited to jails and prisons, have been epicenters for COVID-19 transmission since the earliest days of the COVID-19 pandemic (Hawks et al., 2020; Wurcel et al., 2020). Although less publicized than the impact of COVID-19 in jails and prisons, COVID-19 disrupted the United States (U.S.) trial court system, delaying timely access for tens of thousands of people to a speedy trial (Baldwin et al., 2020; Daftary-Kapur et al., 2021; Draper 2020; McKenna, 2022; Thumma & Reinkensmeyer, 2022). As evident in jails and prison, broadened access to rapid COVID-19 testing has proved to be efficient in reducing COVID-19 transmission both in the carceral setting and the community setting (Drain, 2022; Duarte et al., 2022; Hagan et al., 2021; Mazzilli et al., 2021). Unlike other state and federal locations like schools and carceral settings, (Duarte et al., 2022; Haroz et al., 2022; Qureshi et al., 2022; Schechter-Perkins et al., 2022) trial courts were not a site routinely selected for operationalization of low barrier access to rapid COVID-19 testing. Trial courts are cross-over locations for community members, trial court employees, incarcerated people, and other visitors. Increasing mitigation factors to stop the spread of COVID-19 at trial courts has broad implications for people in the trial court and the surrounding communities.

Massachusetts was one of the earliest and hardest hit states by COVID-19, beginning in March 2020. In April 2020, the Massachusetts Judiciary issued a “gating” strategy with guidelines for trial courts to gradually re-open or scale back operations, if local health data worsened (Courts Suspending Jury Trials as COVID-19 Cases Surge n.d) As part of the Governor’s declared state of emergency, Massachusetts Trial Court physical locations were closed to the public from March 14th to July 13, 2020, except for emergency matters which could not be conducted virtually or telephonically. Trial Court operations shifted to support social distancing, with modifications including adding secure drop boxes at the court for submission of court documents and increasing telephone and computer-based communication. When the MA Trial Court locations re-opened to the public on July 14, 2020, they gradually increased the number of in-person hearings in phases, while continuing to hold various non-emergency matters virtually (Courts, 2022; Gants et al., 2022).

The trial courts represent an important entity in the criminal-legal process that was impacted by COVID-19, but there has been little research on the mitigation strategies operationalized to keep trial courts open (GANTS ICJ. n.d). The goal of this study was to use the Consolidated Framework for Implementation Research (CFIR) and the Expert Recommendations for Implementing Change (ERIC) to design implementation strategies to mitigate the barriers and leverage the facilitators of implementing COVID-19 testing at MA Trial Court locations (Powell et al., 2015).

## Materials and methods

Formation of the Implementation Team: Our pilot project originated from a parent RADx grant with a focus on carceral settings like jails and prisons (RADx: COVID-19 Testing & Prevention in Correctional Settings n.d). Research team members (LBR and AGW) had previously collaborated on projects in jails and prisons and recognized that there was a gap in knowledge about testing in other settings like trial courts (Wurcel et al., 2020). AGW reached out to colleagues working in the U.S. Trial Court system in May 2021, the genesis of further outreach to key stakeholders. In addition to the core Massachusetts research team (AGW, YN, ES, BP), other people on the implementation team included: members of MA Trial Court employees (HR, employee relations, union-representative, and administrators), MA Department of Public Health employees (epidemiologists and data specialists), and representatives from Armstrong Ambulance (the vendor who contracted with the MA Department of Public Health to perform rapid COVID-19 testing). Stakeholders were contacted through email and implementation meetings began in September 2021 and were conducted over Zoom.

Implementation Strategy Selection: During each meeting with the implementation team, the group discussed logistics of the pilot project, such as coordinating shipping BINAX COVID-19 tests to Armstrong Ambulance, creating a testing schedule and working through barriers. Collectively, we decided to pilot offering rapid COVID-19 testing through an ambulance service weekly at one MA Trial Court location. We held interagency workgroup meetings and collected data during those meetings to both identify and work through barriers. Based on previous work with the CFIR, our research team chose to use the ERIC list of strategies, a compilation of 73 implementation strategy terms and definitions, to address the barriers of the pilot project and guide the process of implementation (Powell et al., 2015). The ERIC strategies were selected as barriers arose during the planning and implementation of the project. After identifying barriers, we corresponded them to the CFIR barriers and used the CFIR-ERIC matching tool to guide our selection of implementation strategies from the ERIC list (Consolidated Framework for Implementation Research, 2022; Powell et al., 2015). The research team reviewed all 73 strategies to decide which ones best applied to our research goals and addressed our CFIR barriers.

### Selection of implementation outcomes

We selected adoptability and implementability as the primary implementation outcomes, drawn from the CFIR Outcomes Adendum (Damschroder et al., 2022; Proctor et al., Mar 2011). Adoptability is defined as, “the likelihood key decision-makers will decide to put the innovation in place/innovation deliverers will decide to deliver the innovation’’ (Weiner et al., 2017). Adoption was measured by recording attendance at meetings with the implementation team. Implementability is defined as, “the likelihood the innovation will be put in place or delivered” (Weiner et al., 2017). Implementation was measured as the number of COVID-19 tests performed on testing days. The DPH requested that all rapid test results be reported by uploading a COVID-19 testing template with demographics of the people tested and test results to an online epidemiologic tracking system (Casetivity Webstie n.d). The ambulance company employees filled out this template with identifiable information and uploaded it to the DPH website. Then, they emailed a de-identified excel sheet to the research assistant the week after testing that included the number of tests completed at the site, gender, race, and ethnicity of people being tested, and result of tests.

We aimed to collect end-user (person level, participants, etc.) level feedback on the experience of testing. To understand test-users testing experiences, we chose secondary implementation outcomes of acceptability and feasibility, drawn from work by Proctor et al., and assessed the outcomes using a survey (Proctor et al., 2013). The ambulance company displayed a flyer with a QR code for an anonymous survey consisting of six questions. The first five questions surveyed participants on the feasibility and acceptability of testing at the Trial Court sites using the 5-point Likert scale. Three of the questions were adopted from the Acceptability of Intervention Measure (AIM) and Feasibility of Intervention Measure (FIM) while the other two questions were developed by the research team (Weiner et al., 2017). The last question on the survey asked participants to share their COVID-19 testing experience at the Trial Court in a text entry box (Appendix A, Survey). All participants were 18 years of age or older. Participants were asked for consent and were not compensated for completing the survey. The survey was initiated on the 5^th^ testing date; thus, it was only available at the last four testing sites.

Imbedded within the development of the pilot project and selection of implementation outcomes, we selected “reflection” as a process outcome (Hayashi et al., 2021). Drawn from the processual validity approach framework, reflection is a process outcome that, “includes activities that support the construction of illations through reflection and reflexivity” (Hayashi et al., 2021). Reflection as a process outcome includes peer debriefing and member checks. Each Monday after the scheduled Friday testing date, the implementation team met to reflect on the testing event and prepare for the upcoming week. Additionally, prior to the meetings, the research team analyzed the testing data to share test results with the implementation team.

## Results

### Pilot study roll out

**Fig. 1:**
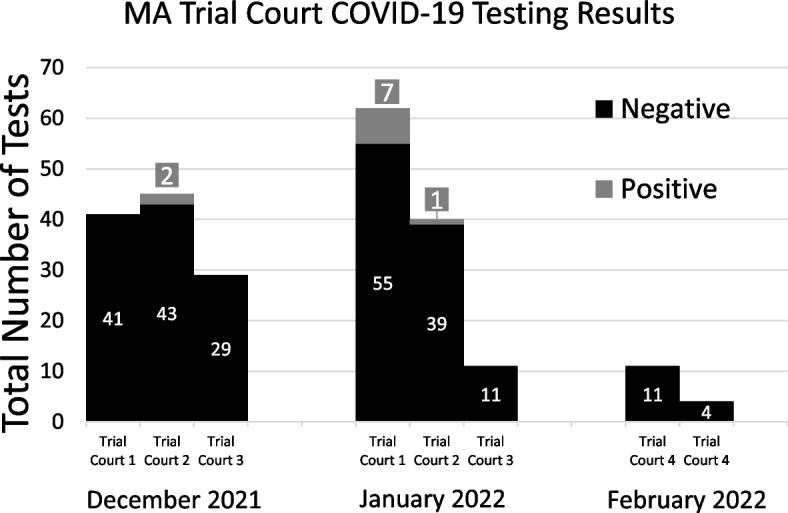
COVID-19 testing results at trial courts in MA

COVID-19 testing pilot project planning took place from May–November 2021, and the pilot project took place from December 2021-February 2022. During preliminary interagency discussions, four Trial Court locations were chosen to increase feasibility of successful implementation. These courts were chosen because relative to the other Trial Court locations, they have the highest number of cases and employ the most people. Testing occurred at each Trial Court location on two dates for a total of eight testing dates. All testing took place on Fridays between 12/1/2022 and 2/18/2022 (Fig. 1).

Implementation of eight ERIC strategies: Barriers that emerged in the process of planning were successfully addressed using ERIC strategies. The team collectively reflected on decisions, with active discussions of pros and cons of potential decisions. Below are examples of decisions made at the implementation team meetings. The eight implementation strategies chosen from the ERIC list are displayed in Table 1 with the associated action steps. A key part of the pilot project development was engaging stakeholders early on the multitude of questions that emerged about details of operationalization. There was considerable deliberation among the implementation team about legal, financial, and operational issues discussed in the planning phase (see below).

1. Location of COVID-19 testing at the trial court: Originally, the team discussed COVID-19 testing inside the Trial Court sites, but after further consideration, there was concern that testing inside courthouses could lead to further transmission. To address safety concerns about COVID-19 testing inside Trial Court locations, the DPH agreed to pay for an outdoor mobile testing unit, operated by a local ambulance company (Armstrong Ambulance). Prior to each testing date, the ambulance company was given instructions on where to park at the Trial Court location and the contact information of an onsite Trial Court employee in case there were any issues. Another operational issue that was discussed was the storage of tests outdoors during the winter. We consulted with experts and confirmed that the validity of the tests would not decrease in cold temperature. Snowstorms caused two testing dates for Trial Court location 4 to be rescheduled to February.
2. Who should be offered testing: There was considerable discussion about the populations (e.g., employees, visitors, and community members) who should have access to COVID-19 testing at the Trial Court. After several discussions, we decided that the testing should be open to Trial Court visitors and employees.
3. Testing Advertising: COVID-19 testing was advertised in MA Trial Courts parking lots and to Trial Court employees via email. The ambulance company displayed ‘Free COVID-19 testing’ signs at the testing sites to direct visitors and employees to the ambulance truck for testing. However, there was no additional advertisement. The implementation team decided to not broadcast testing to the community because testing was only offered twice at each Trial Court location. The question of advertisements was an active point of discussion during meetings. In the end, we felt that advertisement limited to signs at the testing sites and emails sent to the court employees (through the Unions) were the best method.
4. Tracking Test Results: The implementation team discussed how best to deliver results to the person being tested and to track testing both for the DPH as a public health measure and as an implementation outcome of this study. Armstrong Ambulance collected patient demographics and shared the data with the DPH. Then, the ambulance staff de-identified the data and shared it with the research team. The implementation team collectively decided that once a test was complete, the person who was tested would receive the test results on paper within 15 min of testing. The implementation team developed a template for the ambulance staff to record the results and hand to the person, if requested.

**Table 1:** Implementation strategies as applied to COVID-19 testing in trial courts

*Implementation Strategies*	Examples of Application to Trial Court Study
Identify and prepare champions	Identified and contacted key stakeholders; the DPH, trial court employees, and an ambulance company experienced with rapid COVID-19 testing
Fund and contract for clinical innovation	Funding for the COVID-19 tests and research staff time supported by NIH grant
	Funding for the vendor (Ambulance Company) supported by the DPH
Develop academic partnerships	The research team facilitated the process of implementation through organizing meetings, preparing educational material, and facilitating communication between stakeholders
Facilitation	
Assess for readiness and identify barriers and facilitators	There was concern that if people came inside Trial Courts for testing, they could transmit infection. This led to the decision of conducting the testing outside
	As implementation took place in winter, the vendor was concerned about people waiting outside for testing and the accuracy of COVID-19 testing in below freezing temperatures. The ambulance company converted a bus into a testing center
Promote adaptability	There were several snow days, and two testing dates had to be rescheduled
Develop a formal implementation blueprint	With feedback from stakeholders, we created a COVID-19 testing schedule at designated Trial Court sites in MA and reviewed plan with stakeholders
Conduct local needs assessment	Created a QR code survey for participants to share their COVID-19 testing experience at the Trial Court

### Implementation and process outcomes

#### Adoption

The core group of stakeholders involved in the implementation included eleven people. The first stakeholder meeting took place on 9/24/2021. There were six meetings between that date and the completion of testing on 2/18/2022. Eleven people were invited to six meetings from September 2021 to January 2022 (Table 2). Everyone who was invited to the meetings attended at least one meeting. We approached each meeting with a discussion topic such as testing schedule, Trial Court approval, and operationalization of testing. It was important to have all stakeholders present at the meetings to confirm details and advance the conversation. Meeting summaries were e-mailed to stakeholders at the end of each meeting to ensure clarity of plans. In the case that a stakeholder could not attend a meeting, a representative from their organization attended the meeting. Reflection through zoom meetings on Monday about the testing event that occurred the prior Friday was helpful to ensure lines of communication were open. Important topics that came up during the reflection portion included confirming who the ambulance company should contact on arrival to the court, confirming the location that the ambulance should park, and planning for events like snowstorms.

**Table 2:** Adoption outcome 1: attendance of stakeholders at implementation meetings

Meeting date	% Attendance
9/24/2021	82% (9/11)
10/8/2021	58% (7/12)
11/22/2021	80% (8/10)
12/6/2021	80% (8/10)
1/10/2022	50% (5/10)
1/31/2022	70% (7/10)

#### Implementation

A total of 243 individuals were tested: 58% female; 23% White Hispanic, 62% White Non-Hispanic, 13% Black non-Hispanic, 2% Asian Non-Hispanic, and 0.4% American Indian non-Hispanic. There was a 4.1% positivity rate during the testing period (Fig. 1).

#### Acceptability/feasibility

Out of the 66 participants who received a COVID-19 test during the last four testing dates, six participants responded to the QR survey (9% total response rate). All respondents felt that COVID-19 testing at the Trial Court sites was either very acceptable or acceptable. All respondents felt that COVID-19 testing at the Trial Court sites was feasible. Some examples of the responses to the free entry question, “Tell me about your COVID-19 testing experience at the Trial Court today”, included, “quick and simple”, “safe”, “great group of people that work there”, “they are very pleasant and professional”, “easy and fast”, and “the fellows who did the testing were pleasant and knowledgeable. The results came back in less than 15 min. Cannot beat the convenience.” There was no negative feedback shared by participants.

## Discussion

Our study provides a blueprint for how representatives of the trial courts, public health experts, and academics can partner to implement COVID-19 testing. Implementation of COVID-19 testing in MA Trial Courts was feasible through partnership with key stakeholders, facilitated by external funding sources and research collaboration. A crucial element to health services research is gaining the trust of key stakeholders to partake in implementation science. We would like to thank the MA Trial Court representatives and the MA Department of Public Health for their partnership in embarking on this truly innovative research.

As decarceration is a key tenet of the multi-faceted COVID-19 mitigation strategy in carceral settings, keeping trial courts open through waves of the COVID-19 pandemic is crucial (Barsky et al., 2021). An estimated 74% of people in jails are pre-trial—meaning they have not been convicted of any crime (Hawks et al., 2020). Although regional differences in public health mandates and virus transmission patterns differentially impacted courts across the United States, it is hard to imagine that any court system has functioned without COVID-19-related impediments in the past two years. Even with the shift to virtual operations, there were considerable negative socioemotional and financial impacts on people who were detained and awaiting trial (Wiley & Vladeck, 2019). Jurors have expressed anxiety towards going to court during the pandemic (Ma n.d). There is concern that the heightened anxiety may interfere with jurors’ decision-making and focus on the trial (Ma n.d). In addition to vaccination and masking, free access to COVID-19 testing at trial courts can help employees, jurors, people who are involved in litigation, and detainees to feel safer going to court.

We learned several important lessons during the process of developing the pilot project, implementation, analysis, and writing the manuscript. The most important lesson we learned is the importance of clear communication. Although most communication is done over email, the optimal communication was done over the phone or in zoom meetings. Deliberately and intentionally gathering stakeholders’ opinions and approval was necessary before moving forward with decisions. With busy schedules both within and outside of work, sometimes people could not attend meetings. Communication about meetings sometimes required follow-up with stakeholders individually to share the meeting updates and get their approval.

Several limitations need to be considered. Experience in MA may not be generalizable to other states. MA has a higher vaccination rate than other states (See How Vaccinations Are Going in Your Couty and State n.d). We do not know from the data if the people who were tested were employees or people who were visiting the Trial Court as court users involved in criminal or civil matters. At the time of testing, there were policies in place from Trial Court leadership requiring unvaccinated employees to produce a negative test weekly to come to work; therefore, the mobile testing sites may have been more heavily used by employees than by court users. Additionally, we had a low rate of response to the QR-survey, and we were unable to time the initiation of the QR-based survey with the first day of testing because we decided to include the survey after testing had begun. The low rate of the acceptability/feasibility survey could have been a result of suboptimal advertisement, survey fatigue, or concern that the answers to the survey were not confidential. It could also be that participants did not know how to use the QR code or that participants had other competing tasks. There is no data about using QR-based responses in trial courts, thus we hope that our experience can inform future attempts. Finally, we received NIH-funding for this study that supported researcher time and the cost of the tests. Replication of this pilot study may be difficult without financial resources.

Despite these limitations, we are one of the first research teams to report on partnering with stakeholders in a trial court system on COVID-19 testing. COVID-19 testing in trial courts presents an important and underutilized opportunity to protect the health of people living and working inside carceral facilities and their surrounding communities.

## Data Availability

Data sharing is not applicable to this article as no datasets were generated or analyzed during the current study.
